# Stochastic Counterfactual Risk Analysis for the Vulnerability Assessment of Cyber‐Physical Attacks on Electricity Distribution Infrastructure Networks

**DOI:** 10.1111/risa.13291

**Published:** 2019-02-27

**Authors:** Edward J. Oughton, Daniel Ralph, Raghav Pant, Eireann Leverett, Jennifer Copic, Scott Thacker, Rabia Dada, Simon Ruffle, Michelle Tuveson, Jim W Hall

**Affiliations:** ^1^ Environmental Change Institute University of Oxford Oxford UK; ^2^ Cambridge Centre for Risk Studies University of Cambridge Cambridge UK; ^3^ Cambridge Judge Business School University of Cambridge Cambridge UK

**Keywords:** Critical National Infrastructure, cyber‐physical attack, infrastructure

## Abstract

In December 2015, a cyber‐physical attack took place on the Ukrainian electricity distribution network. This is regarded as one of the first cyber‐physical attacks on electricity infrastructure to have led to a substantial power outage and is illustrative of the increasing vulnerability of Critical National Infrastructure to this type of malicious activity. Few data points, coupled with the rapid emergence of cyber phenomena, has held back the development of resilience analytics of cyber‐physical attacks, relative to many other threats. We propose to overcome data limitations by applying stochastic counterfactual risk analysis as part of a new vulnerability assessment framework. The method is developed in the context of the direct and indirect socioeconomic impacts of a Ukrainian‐style cyber‐physical attack taking place on the electricity distribution network serving London and its surrounding regions. A key finding is that if decision‐makers wish to mitigate major population disruptions, then they must invest resources more‐or‐less equally across all substations, to prevent the scaling of a cyber‐physical attack. However, there are some substations associated with higher economic value due to their support of other Critical National Infrastructures assets, which justifies the allocation of additional cyber security investment to reduce the chance of cascading failure. Further cyber‐physical vulnerability research must address the tradeoffs inherent in a system made up of multiple institutions with different strategic risk mitigation objectives and metrics of value, such as governments, infrastructure operators, and commercial consumers of infrastructure services.

## 1. INTRODUCTION

In December 2015, a power outage occurred in the Ukraine (Xiang, Wang, & Liu, 2017), where a Trojan was found on a number of electricity substations believed to be associated with a BlackEnergy Malware campaign utilizing remote cyber intrusion (Sullivan & Kamensky, 2017). This was the first known instance where a cyberattack caused an electricity blackout. Consequently, a key issue is how to develop risk analytics for emerging threats, such as cyber‐physical attacks on energy infrastructure, where we have limited information and data on the level of risk posed. Indeed, since the Ukrainian attack multiple news outlets have reported that malicious software has been found on computers belonging to energy companies in the United States (CNN, 2017; The Washington Post, 2017; USA Today, 2016), raising concerns around the growing vulnerability of Critical National Infrastructure (CNI).

The purpose of moving toward cyber‐physical systems (including sensing, computing, and communication hardware/software) is to develop intelligent monitoring and control of the physical world (Hu, Lu, et al., 2016). However, the continuing shift toward smart cities, smart grids, and the Internet of Things raises issues associated with increased connectivity, and resilience. Indeed, the World Economic Forum's Global Risks Report 2017 ranked the threat of cyberattacks in the top ten for likelihood due to the growing number of physical systems connected to the Internet (World Economic Forum, 2017). Other vulnerabilities include poor cyber security compliance, insufficient institutional training, the use of outdated legacy software, vendor–contractor management practices, and the increasingly easy access to hacking resources.

Yet, one of the largest concerns to defenders is the risk from zero‐day vulnerabilities, on which by definition we do not have existing information, as the first time a vendor is made aware of an exploit is the day an attacker utilizes it. Considerable resources must be placed into identifying and patching the vulnerability by the vendor, which can only begin once the attack has begun.

Due to the nature of rapidly evolving cyber threats, we have still some distance from undertaking effective resilience analytics of cyber‐physical risks. Yet, decision‐makers have highlighted the need for a quantitative framework that shows the direct and indirect socioeconomic impacts of “what if” scenarios. Whereas there are robust event sets of past natural catastrophes (e.g., hurricanes, flooding), we have very limited information for cyberattacks on CNI, including energy, transport, telecommunications, water, and waste, which are studied in this article. We propose to overcome data limitations by applying stochastic counterfactual risk analysis as part of a new vulnerability assessment framework (see Woo, Maynard, & Seria, 2017). This assessment focuses on developing spatial attack footprints in the context of the direct and indirect socioeconomic impacts of a Ukrainian‐style cyber‐physical attack taking place on the electricity distribution network serving London, and surrounding regions in the southeast of England. We apply both downward counterfactual analysis, where we explore the implications of a greater number of substations being affected than the Ukrainian attack, and upward counterfactual analysis, considering what would have happened if fewer substations were affected. When we convert an attack footprint to a consumption shock for economic impact assessment, we assume a 24‐hour blackout.

This counterfactual framework provides a more structured and rigorous approach to the risk analysis of emerging threats by using evidence‐led scenarios, reducing subjectivity in the selection of scenario parameters, by promoting benchmarking and calibration against severity (measured here as the number of electricity customers disrupted). To develop and demonstrate this counterfactual vulnerability assessment for emerging threats, we investigate the following research questions using a cyber‐physical attack on the United Kingdom as a case study:

1. What is the direct impact on power consumers and how does this scale with the number of substations compromised from a cyber‐physical attack?
2. What is the indirect impact of a cyber‐physical attack to other infrastructure users beyond electricity?
3. Does the vulnerability assessment present insights beyond the substation level, toward a systemic understanding of cyber‐physical risk?

Previous research for three different types of electricity blackouts on two different continents has found that it can be incredibly challenging for infrastructure operators to identify their own exposure (Cambridge Centre for Risk Studies, 2015, 2016; Oughton et al., 2016). Although this may suggest a lack of defender capability, the root of the difficulty is that CNI networks have evolved over decades into very large socio‐technical systems comprised of thousands of assets, technologies, networks, and operator protocols. Analytics that show the potential risk to CNI are also required by other parties such as governments, who have a responsibility to protect their citizens, but lack evidence on this matter.

It has been reported that almost half of all U.K. firms have been affected by a cyber breach or attack in 2016–2017 (Department for Culture, Media and Sport, 2017). Consequently, the United Kingdom has made a multi‐billion‐pound commitment to cyber security, as outlined in the National Cyber Security Strategy (HM Government, 2016). Moreover, in the National Risk Register of Civil Emergencies, the U.K. Cabinet Office (2017) identifies both (i) widespread electricity failure, and (ii) the risk of cyberattacks on CNI as having the potential to cause significant disruption. If a widespread electricity failure were to take place, current recovery plans called “Black Start” could take up to five days due to a total or partial shutdown, with some potential disruption beyond this timescale (Cabinet Office, 2017).

The rest of this article is presented as follows. In Section 2, we undertake a literature review before presenting the method in Section 3. The results are reported in Section 4 before being discussed in Section 5. Finally, we conclude in Section 6.

## 2. LITERATURE REVIEW

In this literature review, we first evaluate counterfactual approaches for risk analysis before examining the cyber‐risk assessment of CNI. Finally, the socioeconomic impacts of infrastructure failures are reviewed.

### 2.1. Counterfactual Approaches for Risk Analysis

“Counterfactual” quite literally means *contrary to the facts*, and usually involves a point of departure from a set of historical decisions or outcomes (Roese, 1997). Using counterfactuals as a method of intellectual inquiry is not new, as it has been applied by philosophers and historians for at least two millennia (Tetlock & Belkin, 1996). Although there has been some focus on developing counterfactuals for risk analysis purposes (Bachand, Sulsky, & Curtin, 2017; Holford & Clark, 2012; Jeon et al., 2012; Nassios & Giesecke, 2017), they have not been used to assess vulnerability, or ideally to develop resilience analytics for cyber‐physical risk—an area where there is significant potential for application due to data limitations. Indeed, there are numerous ways that counterfactual analysis could usefully be applied to the science of emerging risks, including modeling of past events using stochastic forensics or scenario event trees (Woo et al., 2017).

Standard critiques of *counterfactual analysis* focus on issues of causality. For example, where both events *c* (the cause) and *e* (the effect) occur, predicated on the assumption that *e* would not occur if *c* did not either (see Collins, Hall, Hall, & Paul, 2004; Lewis, 1974). This critique is commonly applied to the use of counterfactual “what if” questions, such as the manipulation of econometric regression equations to assess economic change. Additionally, there are strong cognitive biases introduced by the analyst about how the system in question functions. In contrast however, *counterfactual risk analysis* recognizes the stochastic nature of history, and the fact that there are numerous alternative realizations of the past. Importantly, the objectives of counterfactual risk analysis are different from counterfactual analysis *per se*, in that the aim is to track potentially catastrophic “black swan” events using the past event catalogue and to provide an increased evidence base for risk‐related decision making.

**Figure 1:**
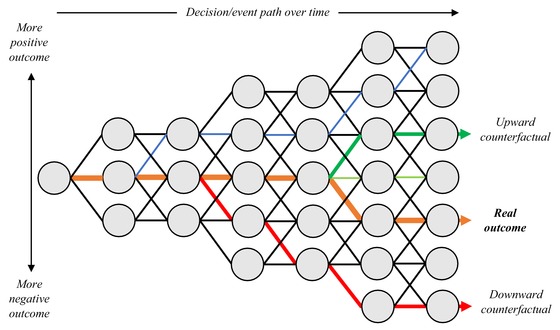
Conceptualization of upward and downward counterfactuals.

After significant disruptive events, we inevitably evaluate mitigation measures and broader resilience strategies, and how they could have reduced the impact, otherwise known as “upward” counterfactual thinking, involving the hypothesizing of how an event could have led to a less negative outcome. While this may be useful for risk practitioners, in contrast, we may rarely ask ourselves whether an event could have been much *worse*, otherwise known as “downward” counterfactual thinking. Fig. 1 illustrates this conceptually, whereby different actions lead to more positive or negative outcomes, allowing a single event to be recreated as either an upward or downward counterfactual. Each node represents a decision in an event path over time, which has the potential to lead to either a more positive or negative outcome.

Cyber risk is a classic example of how “a statistical analysis of past incidents alone does not provide a full description of the risk of future attacks” (Paté‐Cornell, Kuypers, Smith, & Keller, 2017). Indeed, our contention is that counterfactual analysis is a highly appropriate method for assessing cyber‐physical risks to critical infrastructure, as we can borrow and combine information from the number of limited past events as well as from expert elicitation. Consequently, anchoring risk analysis in history helps to mitigate subjective cognitive biases, making the endeavor inherently more plausible and convincing than purely hypothetical scenarios, while still allowing for exploration of unknown exposure via zero‐day vulnerabilities.

### 2.2. Cyber‐Risk Assessments of CNI

While there are many engineering‐focused assessments of direct and indirect interdependencies between power and Information Communication Technologies (Falahati & Fu, 2014; Falahati, Fu, & Wu, 2012), with some focusing on cyberattack risks (Langer, Skopik, Smith, & Kammerstetter, 2016; Ru et al., 2016), we still lack many appropriate methods for identifying key system interdependencies (Oughton, Usher, Tyler, & Hall, 2018). Frameworks for cybersecurity risk assessment and management are increasingly being put forward as existing approaches (including probabilistic and risk‐based decision‐making techniques applied to cyber systems) do not always properly address threats, vulnerabilities, and potential consequences (Ganin et al., 2017). Indeed, qualitative risk metrics are often used within current industry standards for estimating cyber security risk, as opposed to using the quantitative risk metrics commonly found in other industrial sectors, such as finance and banking (e.g., Basel II) (Allodi & Massacci, 2017). Moreover, some assessment methods only focus on existing vulnerabilities, such as the Common Vulnerability Scoring System (Forum of Incident Response and Security Teams, 2017), without putting enough consideration to unknown vulnerabilities. To improve the risk analysis of cyber‐physical systems for CNI, we need not only better cyber vulnerability assessment, but also impact analysis of how vulnerabilities may cause cascading failures to other systems, and ultimately lead to different socioeconomic impacts (Sridhar, Hahn, & Govindarasu, 2012).

One example of a cyberattack on the oil and gas sector in the Gulf Coast of the United States presents a framework for linking cybersecurity metrics to the modeling of macroeconomic interdependencies using the Inoperability Input–Output Model (IIM) (Santos, Haimes, & Lian, 2007). This method has also been used to quantify the risk posed by interdependent Supervisory Control and Data Acquisition (SCADA) systems vulnerable to a cyberattack (Haimes & Chittester, 2005), and fault trees have also been developed to assess the systemic risks associated with cloud computing (Haimes, Horowitz, Guo, Andrijcic, & Bogdanor, 2015). Moreover, one method used to develop effective risk mitigation approaches involves attack and defense modeling to understand the strategic interactions between different agents (Rao et al., 2016; Ten, Manimaran, & Liu, 2010). Increasingly, the risk posed by insider threats has been assessed to provide insight into the human cybersecurity factors affecting an organizations environment during the attack of a corporate cyber network (Gisladottir, Ganin, Keisler, Kepner, & Linkov, 2017). Finally, in an article illustrating multiple approaches, Paté‐Cornell et al. (2017) present a general probabilistic risk analysis framework for the management of critical infrastructure from cyber threats, producing loss results from cyberattacks under different risk mitigation measures. A review of the potential socioeconomic impacts of critical infrastructure failure will now be undertaken.

### 2.3. Socioeconomic Impacts of Infrastructure Failure

Although we do not have many examples of cyberattacks on CNI, we do understand the potential socioeconomic impacts of infrastructure failure due to other natural and man‐made hazards. For example, major blackouts in North America took place in 1996 and 2003 (Anderson, Santos, & Haimes, 2007; Yamashita, Joo, Li, Zhang, & Liu, 2008), as well as in Europe in 2003 and 2006 (van der Vleuten & Lagendijk, 2010), from which we can learn. Also, during the December 2015 flood‐induced outage in Lancashire, United Kingdom, the loss of power in the 132 kV substation led to the loss of supply to 60,987 consumers and complete disruption of digital communications, health care provision, retail businesses and banking, transport, and essential utilities (Kemp, 2016). This happened because this substation was the only grid supply point to the underlying networks of dependent assets. To understand such disruptions across interconnected infrastructure different modeling techniques have been employed, which include, among others, agent‐based models of complex adaptive systems (Schoenwald, Barton, & Ehlen, 2004), empirical analysis (Utne, Hokstad, & Vatn, 2011), system dynamics approaches (Bush et al., 2005), network‐science based models (Zio, 2009), input–output (IO) economics (Oughton, Skelton, Horne, Thomson, & Gaunt, 2017; Santos et al., 2007), macroeconomic econometric methods (Oughton et al., 2019), and computational general equilibrium (CGE) models (Rose, Oladosu, & Liao, 2007; Rose & Wei, 2013). For a detailed literature review on different modeling techniques, see Ouyang (2014).

This article uses infrastructure network models to quantify the social impacts of infrastructure failures, in terms of the metric of customer disruption (detailed explanation is provided in Section 3). Investigating disruptions using this key metric builds on existing analysis of (i) measuring spatial critical hotspots of total disrupted customers affected by failure to England and Wales’ interconnected electricity, transport, water, waste and telecoms infrastructures (Thacker, Barr, Pant, Hall, & Alderson, 2017); (ii) flood vulnerability assessment of electricity networks and dependent water, wastewater, telecoms, and transport assets in the Thames catchment in England (Pant, Thacker, Hall, Alderson, & Barr, 2017); (iii) quantifying daily passenger disruptions on Great Britain's rail network (Pant, Hall, & Blainey, 2016) and (iv) measuring customer disruptions due to flooding and drought exposures of energy, transport, water, and waste networks in China (Hu, Hall, Shi, & Lim, 2016).

The most preferred approaches for measuring the economic impacts of infrastructure failures include the CGE and IO approaches. Rose et al. use CGE models to estimate the business interruption impact of a terrorist attack on the electric power system in Los Angeles, focusing on both indirect economic effects, and the role of resilience (Rose et al., 2007). Port infrastructure has also been the focus for modeling the potential economic consequences of a 90‐day disruption (Rose & Wei, 2013), using a supply‐driven IO modeling approach. The IIM literature includes several studies on economic impact assessment of infrastructure failures (Jonkeren & Giannopoulos, 2014; Santos et al., 2007). Mostly in existing CGE‐ and IO‐based approaches, infrastructures are represented as macroeconomic sectors, with few links established between their socio‐technical network structures and economic characteristics. Recently, studies have established these links, by integrating customer disruptions measured from network failure models with demand‐driven IO models (Pant et al., 2016; Thacker, Kelly, Pant, & Hall, 2018). This article also integrates an infrastructure network model with an economic model, while addressing the shortcomings of existing approaches. For example, many IO models utilize data that are a number of years old due to the complexity and amount of time required to develop national account statistics. Moreover, IO and CGE models can be poor at forecasting future economic states. We overcome this by employing an industry‐standard Macroeconometric Error Correction forecasting model that combines the advantages of both Vector Auto Regression and Dynamic‐Stochastic General Equilibrium methods, allowing the robust testing of scenarios in future periods using the most up‐to‐date information. The method will now be outlined in Section 3.

## 3. METHOD

**Figure 2:**
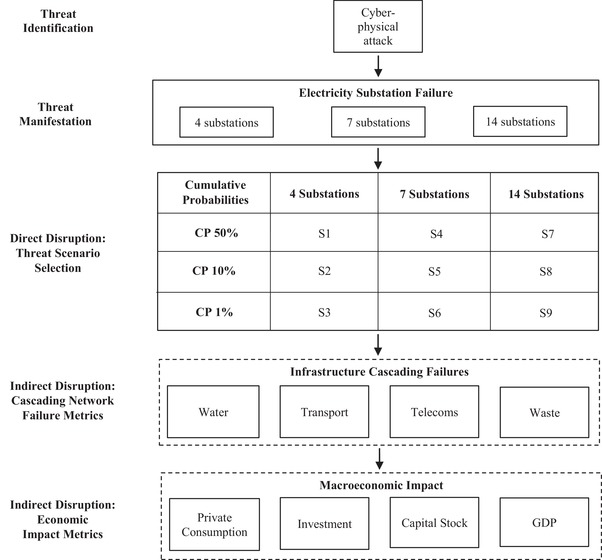
Framework for assessing the socioeconomic impacts of cyber‐physical attack.

We present a framework that allows risk analysts to assess the direct and indirect socioeconomic impacts of a counterfactual cyber‐physical attack on electricity distribution substations. In the following methodological sections, we discuss the specific components of the framework in Fig. 2, including threat identification, manifestation, direct and indirect infrastructure effects, and finally, the macroeconomic impact by scenario. However, we first present a generalized method for quantifying the different components.

As discussed previously, we are interested in counterfactuals of a historic event, *HE*, which we identify as a threat to the system of study. We denote the level of cyber‐physical threat manifestation of the actual event as *HE*_0_, and different counterfactual events as ${\mathrm{HE}}_{1},\ldots ,{\mathrm{HE}}_{z}$. For example, the counterfactual scenario ${\mathrm{HE}}_{i}$ signifies that *m* physical components of an *n* component system could have failed due to a cyberattack. Specifically, in our study ${\mathrm{HE}}_{i}$ signifies that *m* electricity substations out of a *n* substation network could have failed due to a cyberattack. For an exhaustive vulnerability assessment of the counterfactual event ${\mathrm{HE}}_{i}$ we look at all the d=nm combinatorial failure possibilities in the system, to obtain a set of failure scenarios $S_{1},\ldots ,S_{d}$. The failure outcomes are first measured in terms of the populations of direct customer electricity disruptions, $DC_{1},\ldots ,DC_{d}$, corresponding to each failure scenario. Investigating the indirect disruptions for all failure scenarios could be infeasible, given the large number of combinatorial possibilities, so we select certain representative scenarios. This is done by assigning cumulative probabilities $CP_{1},\ldots ,CP_{d}$ to specify the customer disruptions of the failure scenarios. We then sample scenarios based on cumulative probabilities of interest.

Following the selection of a smaller set of specific scenarios $S_{1},\ldots ,S_{h}$, we estimate the indirect customers $IC_{1},\ldots ,IC_{h}$ for different sectors dependent upon electricity. We can also measure the wider macroeconomic impacts in terms of different metrics such as private consumption (PC), investments (In), capital stock (CS), gross value added (*GVA*), or gross domestic product (*GDP*). Hence in the end for a particular counterfactual event ${\mathrm{HE}}_{i}$ we can sample a scenario $S_{j}$, and assemble different direct and indirect metrics $\left(DC_{j},IC_{j},PC_{j},In_{j},CS_{j},GVA_{j},GDP_{j}\right)$.

Fig. 2 represents how the above generalized method was applied in the current study. The initial development of this framework involved a set of stakeholder interviews conducted with representative organizations (number of in parentheses) from energy (13), security (6), insurance (20), defense (2), government (9), and academia (4), to assess current understanding, potential exposure, and analytics which could aid resilience building activities.

Research initially began in July 2015 when defining a hypothetical event similar to the Ukrainian attack, at a set of scenario development workshops, consisting of U.K. representatives from government (5), academia (8), and the electricity (2), defense (1), risk management (3), and cyber security (1) industries (number of in parentheses). Rather than regional control rooms being of greatest risk, which have significant cyber and physical security procedures, it was local substations that were identified as vulnerable assets. Traditionally, these local substations are less protected than those parts of the network which are higher up the distribution (or transmission) hierarchy. While stakeholders identified that pinpointing vulnerable substations was highly challenging, there was specific interest in understanding the potential direct and indirect impacts of different events based on differentiated a priori levels of cyber vulnerability. In December 2015, as this research was being written up into an industry white paper (Cambridge Centre for Risk Studies, 2016), the Ukrainian electricity substation attack took place, turning a hypothetical scenario into an actual event.

### 3.1. Threat Identification

A *threat* is defined as any potential hazard implemented for malicious intent, which could interfere with normal operational conditions, causing a blackout event. This likely involves “hacking” Industrial Control Systems (ICS), which include SCADA systems, distributed control systems, and programmable logic controllers. These systems are found in many industrial applications including CNI systems. Indeed, electricity distribution operators are increasingly using some form of ICS to control, automate, and maintain operation of their equipment, and the flow of electricity (Sridhar et al., 2012). Since Stuxnet sabotaged Iran's nuclear programme in 2009 and 2010, we have seen a dramatic increase in the threat to ICS and SCADA systems, raising concerns around our ability to protect CNI, for example, the Dragonfly Campaign (Symantec, 2017). The attacker may specifically aim to spoof sensors with false data, disconnect key devices required for normal operations, and control physical components such as actuators. As well as remote attacks, there is also potential for intruders to physically connect a rogue hardware attack platforms into the Local Area Network of a substation, breaching “air‐gapped” networks (see Cambridge Centre for Risk Studies, 2016). Having defined the threat, we will now discuss how it manifests.

### 3.2. Threat Manifestation

The 2015 Ukrainian attack has been documented by the U.S. Department for Homeland Security (2016). The attackers managed to firstly deliver BlackEnergy Malware via spear phishing emails using malicious Microsoft Office attachments. Secondly, intruders conducted comprehensive reconnaissance of critical systems in advance. Finally, to commence the attack, substation breakers were disconnected using legitimate credentials with either remote administration tools or remote ICS client software via a Virtual Private Network connection. Little about this attack was specific to Ukrainian technology or critical infrastructure, and therefore it could be replicated in similar ways in other nations. Analysis of this attack (Sullivan & Kamensky, 2017) indicates that (i) the event could have been far worse, and (ii) the idea of a major cyber‐physical attack on energy infrastructure is not a matter of *if*, but *when*.

Although there has been some discrepancy in the number of substations affected by the Ukrainian attack in 2015, approximately 30 substations were affected (Sullivan & Kamensky, 2017), consisting of seven 110 kV and the remainder 35 kV (Electricity Information Sharing and Analysis Center, 2016). However, more substations could have been affected. We replicate the Ukrainian‐style threat for the United Kingdom, which is divided into electricity regions consisting of nine Distribution Network Operators (DNOs), out of which we have chosen one. All substation assets in this electricity region are owned and operated by the same DNO. To capture both upward and downward counterfactual events, we select both half and double the number of substations affected by the Ukrainian attack. We therefore test the implications of four, seven, and 14 substations being compromised by a zero‐day vulnerability. As consequently explained in this method, each of these three potential events is investigated separately utilizing Monte Carlo simulation. The number of substations is used to represent different scales of attack. As this analysis focuses specifically on modeling the socioeconomic impact of a Ukrainian‐style cyber‐physical attack on the U.K., we measure the direct disruption as the proportion of the local population disconnected from the electricity network.

### 3.3. Direct Disruption: Threat Scenario Selection

**Figure 3:**
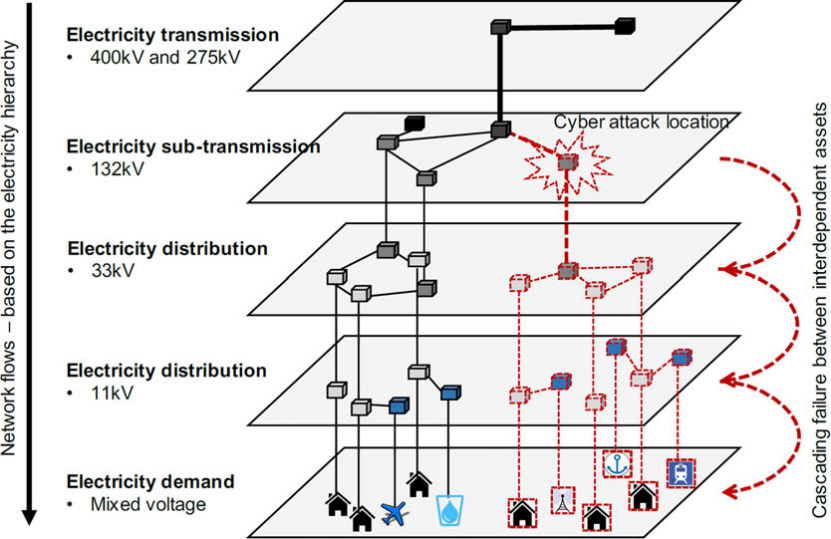
Generalized overview of electricity transmission and distribution in England.

Following the creation of the threat scenarios, we estimate the disruptive consequences associated with different cyber‐physical attacks on electricity substations. Fig. 3 provides a generalized overview of the electricity transmission and distribution networks, which form a hierarchy of flows, from power generation to electricity users, where the electricity is sequentially stepped down from high‐voltage transmission networks to low voltage distribution networks (Thacker et al., 2017). Background information on the U.K.’s electricity network can be found in a briefing by the Parliamentary Office for Science and Technology (2001). “Stepping‐down” is performed by electricity transformers that are located within substations, which we assume are ICS operated, and potentially vulnerable to a remote cyber‐physical attack. Our study focuses on 132 kV substations in the network hierarchy as the points of cyber‐physical attack. To understand the impact, this modeling approach assigns spatially located customers to local distribution substation assets, leading to variation in the number of customers per substation (Pant et al., 2016; Thacker, Barr, et al., 2017; Thacker, Pant, et al., 2017) (further details in Section 3.4), hence leading to “larger” or “smaller” substations. Network topologies for electricity distribution and all other infrastructure systems were meticulously constructed and validated, as detailed in the associated Supporting Information.

Classical scenario analysis is a tool frequently used for risk management purposes; however, it often is used in a very deterministic manner. It is arguably more beneficial to know something about the distribution of possibilities associated with a threat, given that there are a wide range of potential outcomes that could arise (particularly for zero‐day vulnerability risks). There are approximately two hundred and fifty‐two 132 kV substations in the London, southeast, and east of England regions available for the simulation in this analysis. We stochastically explore the event space by randomly sampling four, seven, or 14 substations per event, leading to a total number of 1.6 × 10^8^, 1.2 × 10^13^, 3.3 × 10^22^ substation combinations respectively. Each substation has an equal likelihood of being selected.

This simple approach, in which we sample subsets of affected substations and calculate the population impacted in each case, is justified on two grounds. First, we lack the technical design and operational details of individual components within each substation (e.g., transformer details, switchboards, bus bars, circuit breakers, conductors and insulators, capacitors, etc.), and their potential design fragilities, as well as the actual master‐planning of the substations that would be needed to undertake a (more sophisticated) operational view of impairment. This is partly reflective of the variation in the state of knowledge of CNI operators. But it is safe to assume that all substations of the same DNO function at similar design and operational standards (U.K. Power Networks, 2006). Second, the level of uncertainty embodied in the assessment of cyber‐physical risks to CNI suggests that the uncertainties from zero‐day vulnerabilities will outweigh any additional resolution obtained at the engineering level.

**Figure 4:**
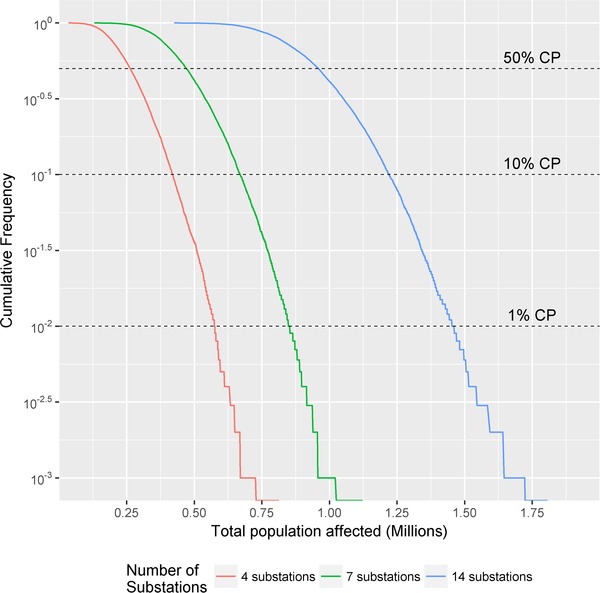
Distribution of direct disruption: Population affected for each stochastic event.

Once we have a data set of simulation results we calculate an *F‐N* cumulative frequency curve for each scenario, where *F* is the frequency attributed to each event *i* based on the number of substations randomly attacked and *N* is the number of people estimated to be affected by each event as follows:

(1) $$\;F_{i}=\;\frac{m_{i}}{\left(n_{i}+1\right)},$$

where $m_{i}$ is the rank of the total population affected by each simulation iteration (with 1 being given to the largest possible value), and $\;n_{i}$ being the total number of simulation runs. Event simulation results are illustrated in Fig. 4, for example, for an event with seven attacked substations, the probability of more than 0.5 million people being affected is approximately 10^−0.3^, hence 50%.

Spatial and indirect disruption results can be found in Section 4.

For a given stochastic event, for example, where seven substations are attacked, the difference in the population affected by the substation selection relates to changes in the spatial attack footprint and the network topology. In contrast, differences between stochastic events relate to the scale of attack across the network and the number of infected substations. We select cumulative frequencies that relate to the mean impact (50th percentile, or median) and the tail risk (10th and 1st percentile), as these were identified as being important to the scenario stakeholders at the development workshops. Tail risk has also been identified as vital in the cyber‐risk literature (Paté‐Cornell et al., 2017).

Fig. 4 shows that an extreme scenario (10th or 1st percentile) associated with a lower number of infected substations, has a smaller disruptive impact than the mean scenario of an event with more infected substations. For example, the 1st percentile of the event with seven infected substations affects power supply for 0.8 million people, while the mean number of disconnections for a 14‐substation event is almost 1 million. The implication for the management of power distribution networks is evident: Preventing (or recovering from) cyberattacks, which can scale up from a few substations to many is the priority. By implication, protecting larger substations with greater investment of resources may be a less effective means of prevention according to the metric of consumer disruption.

### 3.4. Indirect Disruption: Cascading Network Failure Metrics

We also examine other critical infrastructures, including rail, fresh water, wastewater, and telecommunications, which typically derive their electrical power needs from a direct connection to the distribution network. The electricity network is connected to other infrastructures, to map their dependencies on electricity, to create a system‐of‐systems network model with results reported for 379 Local Authority Districts (Thacker et al., 2017). The dependent infrastructures include water towers, wastewater treatment works, macrocellular basestations as point assets, along with a railway network. Fig. 3 already showed a graphical representation of the infrastructure types connected to the electricity network in the demand layer. For details of these infrastructure assets and networks, along with their dependencies, see previous studies (Pant et al., 2016, 2017; Thacker et al., 2017). The dependencies are derived based on multiple criteria including (i) existing data on the physical connections between networked assets; (ii) geographic proximity of assets to their nearest electricity substations of appropriate voltage, and (iii) functional understanding of the flow of electricity from substations to other infrastructures. For example, we consider instances of network redundancies where some large assets can be connected to multiple substations (Thacker et al., 2017), and several railway stations are connected to sets of substations (Pant et al., 2016). The exact voltage level to which individual CNI assets are connected may vary. However, the majority of each asset type connects to the same voltage level (the same substation type).

We define *failure* as a condition of the network node (or edge) asset such that it is no longer able to perform its functional purpose. In our description, this means that the service demand satisfied by the affected node is lost and all its connections are interrupted. Based on the selected scenarios, it is assumed that all the included electricity substations have failed and subsequently the number of disrupted electricity customers is estimated. In parallel there are also cascading failures toward the dependent assets of other sectors.

To estimate disruption, we first model customer assignments to different types of infrastructures. For all infrastructures, we create average daily customer estimates. Electricity, water, wastewater, and telecoms provide utility services over fixed areas called asset footprints (Pant et al., 2016; Thacker, Barr, et al., 2017; Thacker, Pant, et al., 2017). These assets footprints are derived using a Voronoi decomposition technique, which results in connecting customers to their nearest assets in space—an assumption that holds true in the real world (Pant et al., 2016; Thacker, Barr, et al., 2017; Thacker, Pant, et al., 2017). Assigning customer values to assets is based on a spatial union of its asset footprint with census derived population estimates. For the railway network, customer demands are derived using a model of station entries, interchanges, and exits, and by combining these with train frequencies along routes, to obtain daily origin–destination trip assignments for passenger flows (Pant et al., 2016).

For each threat scenario, the infected electricity substations lead to failure propagating along the whole network path where the flow of electricity via the failed substations takes place (as illustrated in Fig. 3). For other critical infrastructures, such as telecoms masts, water towers, and waste water treatment works, customer disruptions are estimated based on whether the connected electricity substation has failed. For the railway network disruptions, we first consider the stations disrupted due to connection to failed electricity substations. Following which we consider all origin–destination journeys that are lost even after rerouting due to disruption of the selected stations (Pant et al., 2016). The aggregated number of customers affected by each critical infrastructure sector provides the disruption estimates reported.

### 3.5. Indirect Disruption: Economic Impact Metrics

We quantify economic impact given a demand‐side economic shock due to reduced private consumption, from households being without power. Private consumption is affected as consumers are unable to complete daily economic transactions. The Oxford Economics Global Economic Model is utilized, which is a widely employed macroeconomic model with users including the International Monetary Fund and World Bank (Oxford Economics, 2017), and consists of over 26,500 interlinked equations based on historical correlations and economic theory. Multivariate forecasts are produced for many economies, but here we focus only on the United Kingdom. The modeling approach adopts Keynesian principles in the short run, where shocks to demand generate economic cycles that can be influenced by fiscal and monetary policy. While over the long run, output is determined by supply‐side factors including investment, demographics, labor, and productivity. The basic modeling principle of the simulation framework is that GDP output (Y) can be expressed as the summation of aggregate Consumption (C), Investment (I), Government Spending (G), and Net Exports (NX) (hence, Y = C + I + G + NX).

We use the model to see the effect of a shock directly applied to private consumption to understand the impact on GDP. A quarterly shock is parameterized using the total population affected for each scenario for a single 24‐hour period. Additionally, we report a set of intermediate macroeconomic indicators including lost investment, capital stock formation, manufacturing GVA, and services GVA, to quantify the economic impact by scenario.

On one hand, this may overestimate the impact due to the potential rescheduling of consumption purchases. While on the other, we do not consider potentially much larger impacts on the supply‐side due to business interruption, or indeed harm to labor productivity. Given the challenge of estimating supply‐side impacts, they are out of scope for this analysis and are identified as an area of future research in the discussion.

## 4. RESULTS

The modeled electricity distribution network serves just under a third of the total U.K. population. In this section, the results are reported for the nine scenarios tested within the assessment framework with a spatial resolution of 379 Local Authority Districts. First, the spatial direct disruption to electricity users will be reported, followed by the indirect impacts. The latter includes spatial customer disruption on non‐electricity CNI and estimates of economic loss due to the consequent disruption to private consumption across all affected infrastructures.

### 4.1. Direct Disruption

**Figure 5:**
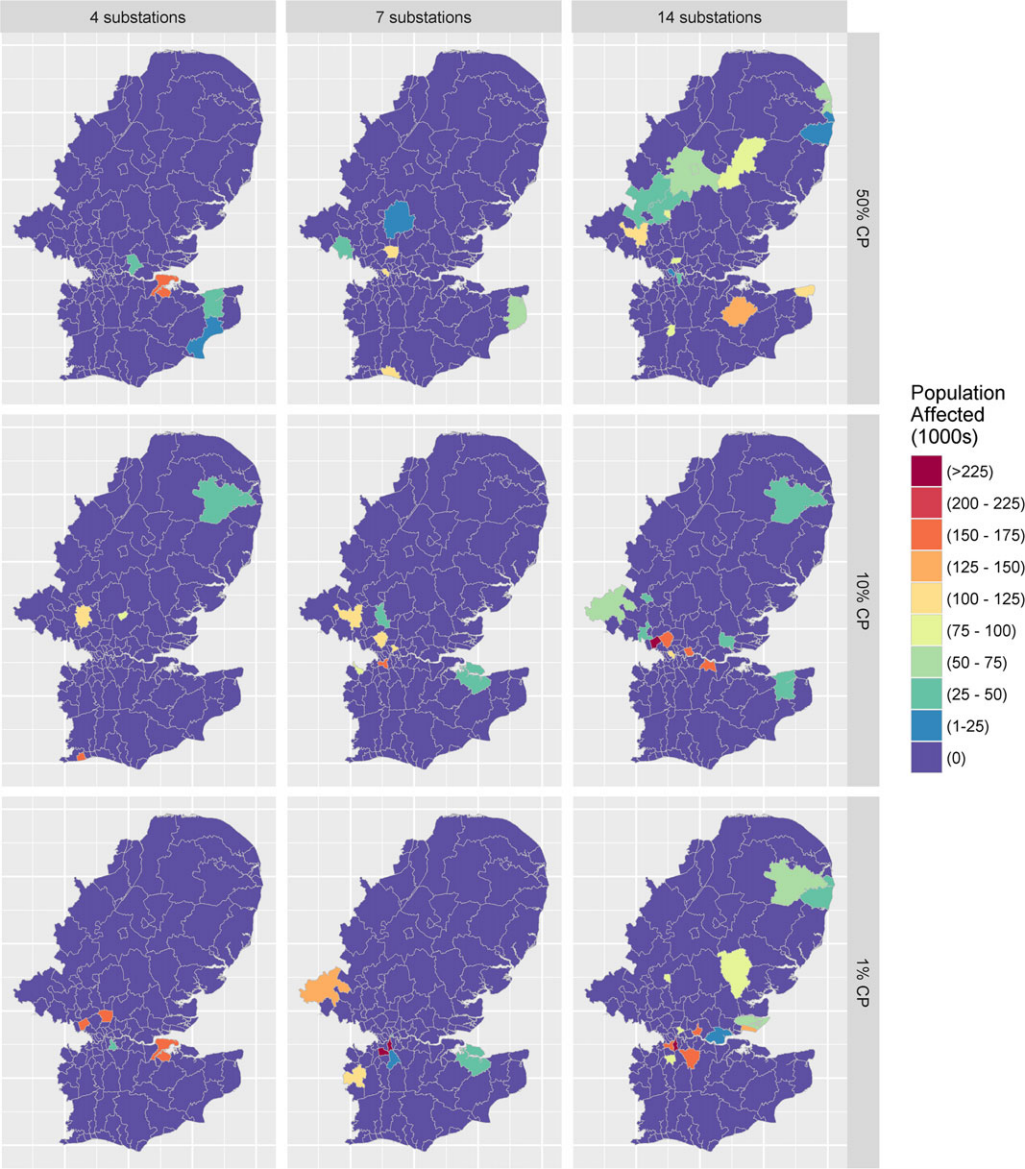
Direct disruption: Spatial impacts of population affect by electricity blackouts.

In the selected scenarios, the population affected ranged from 0.25 to 1.45 million, reflecting between 0.4% and 2.2% of the total U.K. population. A larger proportion was affected as the number of compromised substations increased. In general, as the number of substations affected increased, the matter of which specific substations are attacked has a marginally diminishing impact on the direct level of population disruption. This pattern is illustrated within Fig. 5, where a seven‐substation attack with a 1% probability had a lower severity, in comparison with a 14‐substation attack with only the mean probability (50%).

The spatial attack footprint is visibly different for the seven‐substation and 14‐substation event types, in terms of the scale of the disruption (but less so for four‐substation and seven‐substation event types). However, even in the least severe scenarios, there was still a significant proportion of the population disrupted, with many Local Authorities experiencing between 50,000 and 200,000 people being affected. Having examined the spatial footprints of the direct population impacts, the indirect results will now be reported.

### 4.2. Indirect Disruption

**Figure 6:**
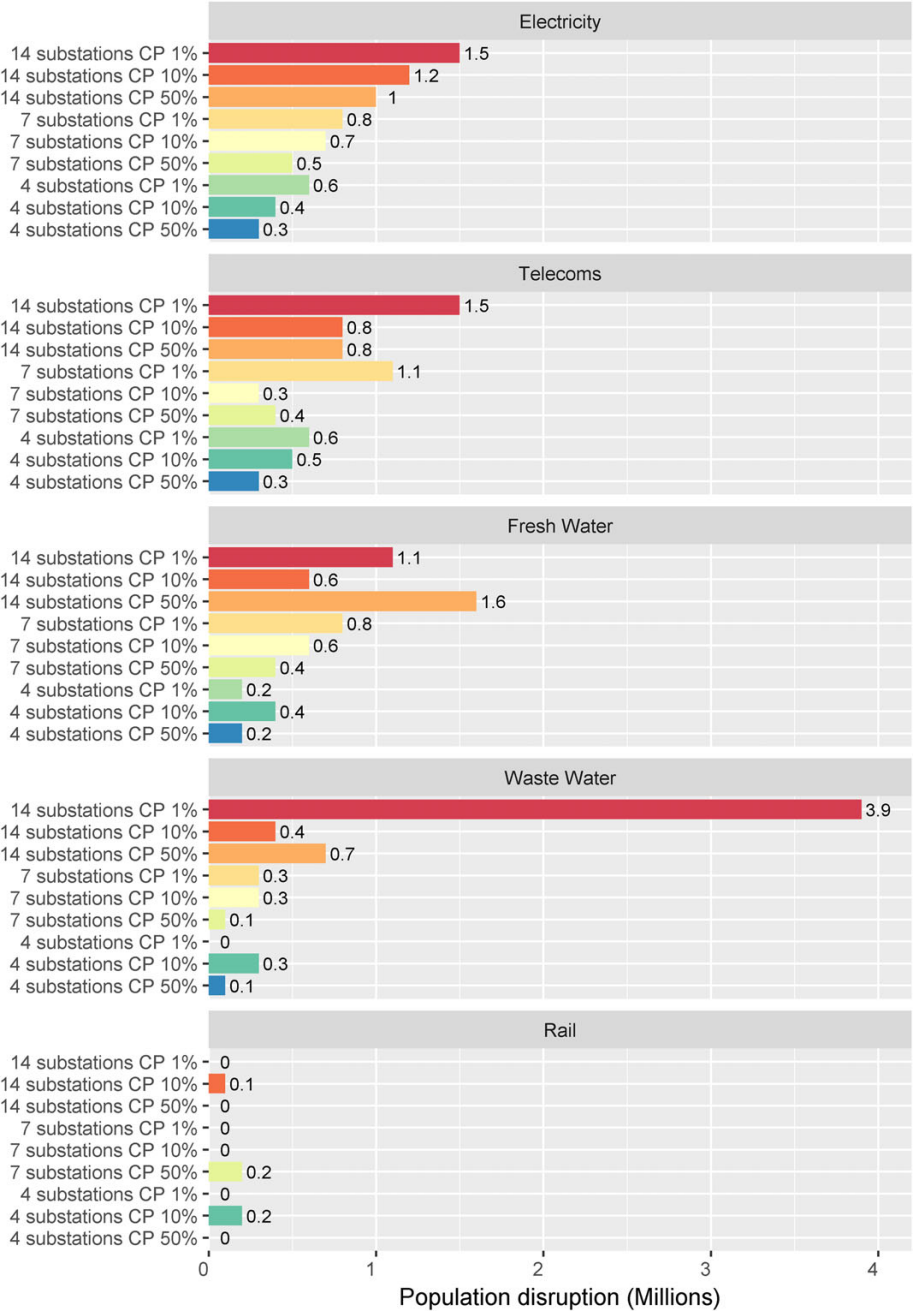
Total direct and indirect disruption by CNI sector.

In addition to direct infrastructure disruption, a cyber‐physical attack on electricity distribution substations could lead to further indirect infrastructure cascading failure. The total direct and indirect population disruption is visualized in Fig. 6, for CNI sectors, allowing evaluation of cascade impacts. The indirect disruption varies by sector and can be less predictable than the direct electricity disruption level. For example, the degree of electricity disruption is well correlated with the number of substations attacked, whereas there is greater variability in rail or waste water depending on whether critical assets are in the affected zone. In general, population disruption ranged from 0.3 to 1.5 million for telecommunications and 0.2 to 1.1 million for fresh water. Greater differences were found in waste water, where the largest user disruption ranged from 0 to 3.9 million, contrasting with smaller levels of disruption in rail (0–0.2 million).

**Figure 7:**
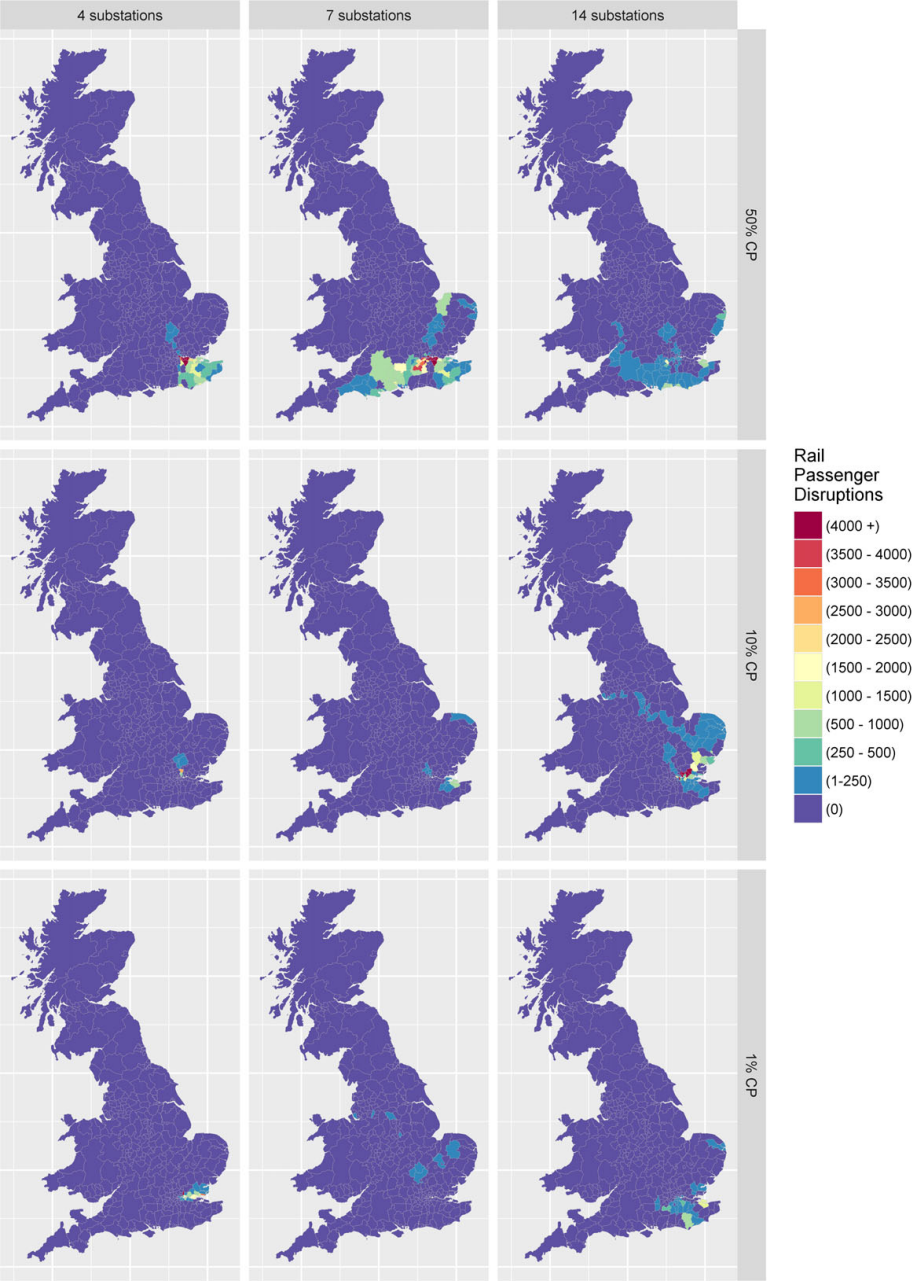
Indirect disruption: Rail passenger disruptions.

Some of the negative impacts would take place outside of the attack zone footprint, with rail disruption again being a prime example, as illustrated in Fig. 7. In most scenarios explored, the biggest impacts were evident in London due to the large number of commuters relying on transportation from suburban or rural locations, into urban areas. However, the spatial distribution of passenger disruptions is highly dependent on the scenario and the location of compromised substations. For example, the seven‐substation event with a 50% cumulative probability leads to significant passenger disruption taking place to the north, west, and southeast of London. Contrary to expectations, passenger disruption was often higher in the scenarios with a 50% cumulative probability, rather than in those with a lower cumulative probability. This results from the cumulative probability being calculated using direct electricity customer disruptions, hence cascading disruption outcomes are consequences of the random substation selection process.

**Figure 8:**
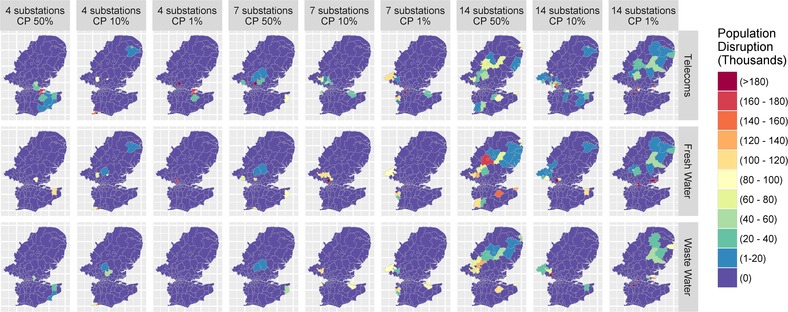
Indirect disruption: Cascading failure across CNI.

The spatial distribution of disruption to other critical infrastructures was less spread across the United Kingdom, as provision is more local. For example, with telecommunications, fresh water, and waste water, services are distributed closer to the end user's premises, mainly confining disruption to the areas with compromised substations, as illustrated in Fig. 8. Hence, the underlying topological structure of the infrastructure network has a significant impact on the spatial extent of disruption. The significance of this disruption for each CNI may manifest in different ways. For example, wireless telecommunications in theory allow users more flexibility to access different basestations, but recent evidence has suggested that during power loss it takes only a couple of hours for the system to become completely inoperable in the blackout zone (Kemp, 2016).

In general, the spatial impact of disruption does not display any obvious pattern. Cascades mainly take place in peripheral (suburban and rural) areas in less severe scenarios (e.g., four substations with 50% cumulative probability), whereas urban locations are visible in severe scenarios (e.g., 14 substations with 1% cumulative probability), with several central London areas affected such as Newham, Westminster, and Greenwich. These results are sensitive to the fact that urban substations have a higher number of customer connections compared to rural substations. Hence, the scenario differences due to cumulative probability display this difference. Importantly, the level of disruption from 14 substations and a 50% cumulative probability, saw larger indirect cascading failure in some sectors (fresh water and waste water) than more severe scenarios, such as 14 substations with a 10% cumulative probability.

**Figure 9:**
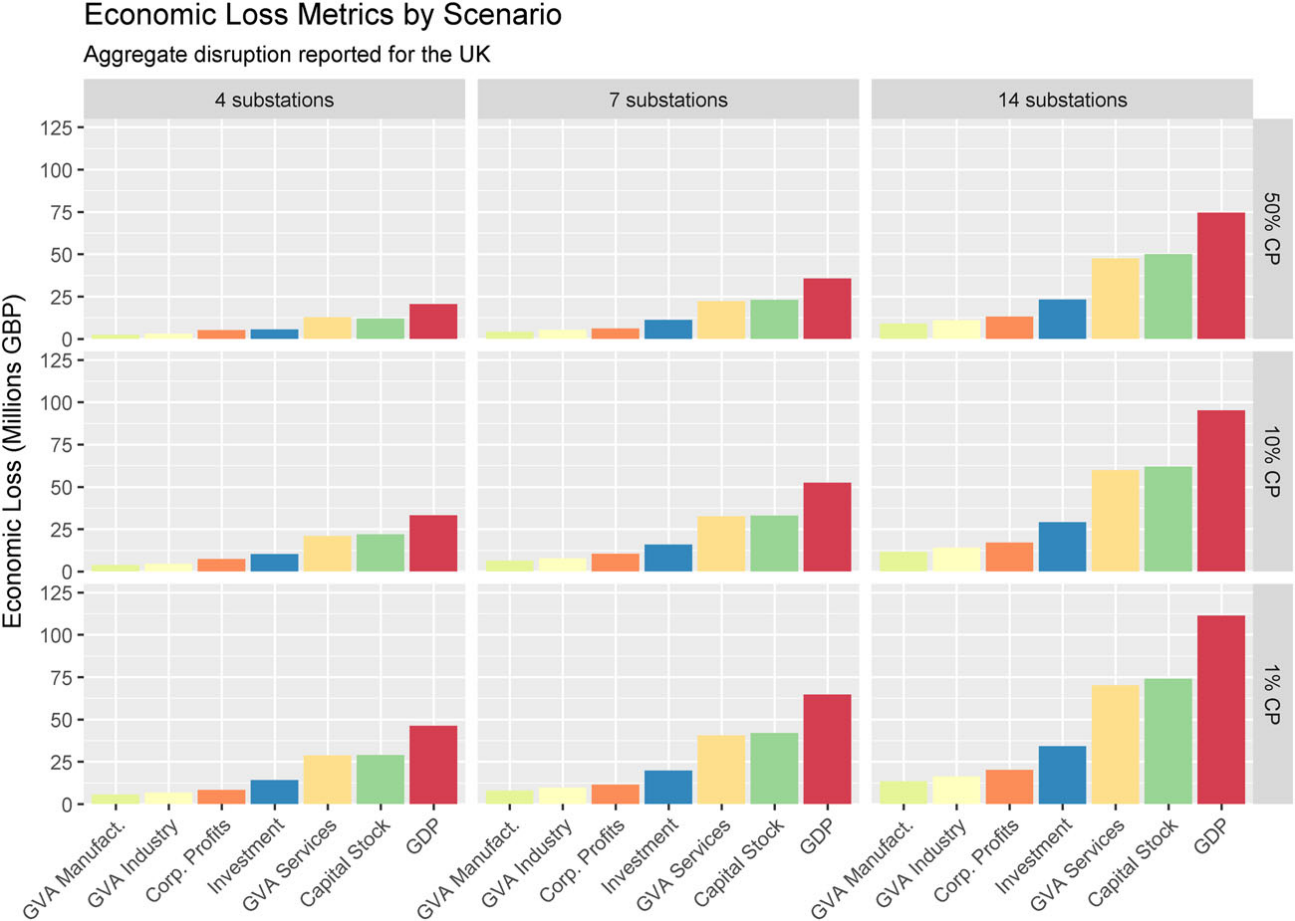
Total impacts by scenario.

The economic impact (see Fig. 9) is assessed based on disruption to consumption, labor supply, and business confidence to obtain a 2018 GDP loss estimate following an event taking place in Q1 2018. The GDP loss ranged from £20.6 million in a four‐substation event with a 50% cumulative probability, up to £111.4 million for a 14‐substation event with a 1% cumulative probability. As illustrated in Fig. 9, the quantity of lost investment and capital stock formation in each scenario was proportionally similar. Lost investment ranged from £6 to 34 million across the scenarios, whereas lost capital stock formation ranged from £12 to 74 million. The GVA impacts broadly reflected the underlying economic structure of the UK's economy, which is dominated by service sector activities. This meant manufacturing GVA loss ranging from £3 to 13 million was relatively minor in comparison with the £13 to 70 million loss range in services.

Having reported the results, we will now discuss their implications in Section 5.

## 5. DISCUSSION

Even the most diligent operators are exposed to zero‐day exploits; indeed, the discovery and application (by attackers) of zero‐day flaws is stochastic by nature. Modeling and pinpointing these exposures is therefore extremely challenging. In this article, the analysis focused on applying counterfactual information to develop upward and downward scenarios of physical asset failure. This enabled us to answer “what if” questions by quantifying impacts without getting mired in specific details of either the attackers’ *modus operandi* or the engineering specifications of the system attacked (which may be unknown or unavailable, respectively). An advantage of the counterfactual risk analysis approach, whereby past events are used as a basis to provide decision‐support evidence on potential future events, is that it limits the influence of researcher cognitive biases when compared to hypothetical scenario risk analysis.

A key finding identified within this article, pertaining to research question 1, is that the size of direct population disruption from a substation attack is better predicted by the number of substations affected, rather than by taking into account the size of population served by (larger) substations. The first order implication is that monitoring and response preparedness across the entirety of substations under the control of a DNO is more valuable than increasing the resistance to attack of more important substations.

Regarding the indirect impacts, pertaining to research question 2, we find that customer disruption for different CNI sectors is correlated with power loss in two distinct ways. For example, telecoms, fresh water, and waste water services are highly correlated with the spatial footprint of electricity disruption. In contrast, in transportation there are relatively few, yet very important, critical hubs. Therefore, there may be a lower probability of infecting a substation which serves one of these critical hubs. However, if this happens, there could be higher disruptive consequences from a single asset. This results from differences based on disruption metrics, and whether the number of direct connections lost (as applied here) or a weighted metric reflecting the relative importance of each connection is utilized (an area for future analysis). For example, airports and maritime ports may deserve a higher weighting due to the economic disruption associated with their inoperability.

The analytics provide the following systemic information, helping to address research question 3. Customer disruptions are highly correlated in some CNI sectors with the spatial attack footprint on the electricity distribution network. However, for other CNI sectors with critical hubs, such as commercial transportation, customer disruption is not necessarily an effective measure of societal value. Indeed, there may be three competing interests for CNI operators. First, meeting regulated service standards for domestic consumers. Second, meeting industrial demand from high‐value customers who purchase large quantities of services with high reliability requirements. Third, providing security of supply for critical infrastructure assets including essential government services, hospitals, healthcare, and commercial information technology for banking and payment services. We believe the next steps toward cyber‐physical resilience analytics will explicitly address the multiple objectives of these competing interests to quantify tradeoffs at the operator and policy levels.

We bring the article to a close with a reflection, aimed at CNI operators. It is difficult to completely disable large numbers of substations, that is, scale an attack, as vulnerabilities are specific to types of substation hardware, software, and all the different components of security that need to be overcome to enable a plan to succeed. Therefore, the greater *diversity* between substations in terms of their software and hardware subsystems, the more difficult it is for a vulnerability of a subsystem to cause a problem at scale (across substations). Indeed, the scalability of the attack depends on the standardization of components and systems in place.

Yet, prescribing a multiplicity of different configurations, such as substation design, subsystems, and implementation, may not be practical, as any increase in the number of configurations requires a comparative increase in resource for maintenance and security. Due to the uncertainty associated with the dynamic nature of cyber, a more appropriate strategy may be to invest in, and actively undertake, comprehensive and widespread monitoring of assets. This approach does not interfere with existing ICS, and will allow a much more efficient response if, or rather when, an attack does take place.

## 6. CONCLUSION

A key finding identified within this article is that the size of direct population disruption from a substation attack is better predicted by the number of substations affected. The number of customer connections at each substation is a less important factor in predicting direct population disruption. This finding was established by testing scenarios which targeted different numbers of substations (four, seven, and 14) and different severity levels (50%, 10%, and 1% cumulative probability). Nevertheless, certain substations are critical for the functionality of other key assets belonging to the railway network or fresh water distribution system, for which other metrics of societal value, other than population disruption, are appropriate.

With such a small history of known cyber‐physical attacks, individual organizations have struggled to justify investment in ICS cyber security measures using traditional return‐on‐investment thinking. We are unlikely to obtain a robust event set of cyber‐physical attacks on CNI in the near future because of the rapidly changing landscape associated with this threat. This is common with low‐probability, high‐impact events, which is a key justification for using a counterfactual approach.

In many countries, such as the United States or the United Kingdom, governments do not own the infrastructure—private operators do. Yet, the public will look to governments when we see another cyber‐physical attack on critical infrastructure. The scenarios presented within this analysis provide further evidence on a developing area, for key private and governmental stakeholders, on how direct and indirect customer disruption takes place for different scales of cyber‐physical attack. This article sets a direction for the assessment of risks that are both systemic and emergent, by undertaking a vulnerability assessment using a stochastic counterfactual risk framework. Cyber‐physical vulnerability assessment, as a path to developing effective resilience analytics, must quantify the tradeoffs inherent in a system made up of multiple institutions with different objectives, such as governments, infrastructure operators, and commercial consumers of infrastructure services.

## ACKNOLEDGMENTS

EO, RP, ST, and JH were supported by the Engineering and Physical Sciences Research Council via the program grant Multi‐scale Infrastructure Systems Analytics (Mistral) (Grant no. EP/N017064/1). The authors would like to thank Lockheed Martin for providing initial research funding to explore the topic. They also thank all participants of a workshop held at the Cambridge Judge Business School in 2015 who provided invaluable input. Arjun Mahalingam provided useful research assistance. The authors have no conflict of interest. The research materials supporting this publication can be accessed from Ordnance Survey and UKPN Regional Development Plans.

## Supporting information


**Fig. S1**. GIS representation of the point assets and the count of the number of different infrastructure asset types.
**Fig. S2**. Visual representation of example dependencies with respect to electricity for other infrastructures.
**Table S1**. Description of the network data used in the study.
