# Supplementary material for: Stochastic Counterfactual Risk Analysis for the Vulnerability Assessment of Cyber‐Physical Attacks on Electricity Distribution Infrastructure Networks
Source: Risk Anal. 2019 Feb 27;39(9):2012–31. doi: 10.1111/risa.13291 (PMC6850035; doi:10.1111/risa.13291)
Supplement: Supplementary file 1 — Fig. S1. GIS representation of the point assets and the count of the number of different infrastructure asset types. Fig. S2. Visual representation of example dependencies with respect to electricity for other infrastructures. Table S1. Description of the network data used in the study. [file RISA-39-2012-s001.docx]

**Description of infrastructure network data**

As described in the paper, the study area is the London, South East and East of England region (henceforth referred to as South-East England). The justification for this choice is that a single region allows greater expert judgement and validation of the constructed network topologies, compared to a large-scale national model. The electricity infrastructure considered within this area are electricity substations operated by a single Distribution Network Operator (DNO). Other infrastructures considered dependent upon electricity within the area include water towers, wastewater treatment works and macrocellular basestations. The railway infrastructure, which is represented for the whole of Great Britain, is also considered as an electricity dependent system through the railway stations connected to the electricity substations in the South-East England region.

For creating the infrastructure network representations, assets such as the electricity substations are represented as points (nodes) and the connectivity between assets is represented as line elements (edges). The geospatial nodes and edge information is derived mostly from GIS data sources in the first instance. The first two columns of Table S-I describe the GIS data assembled for this study.

For each infrastructure type customer demand numbers are estimated in different ways depending upon the availability of data. As described in the paper, the customer numbers for the utility assets (electricity substations, water towers, wastewater treatment works, macrocellular basestations) are derived by combining the census population density maps with Voronoi polygon footprints of these assets. Voronoi polygons create footprints that match population concentrations to their nearest utility asset, which is a rational assumption reflective of our discussions with network asset owners and operators about the way distribution and lower level assets provide services (S. Thacker, Pant, & Hall, 2017). This results from the fact that infrastructure networks in England are radially deployed due to very high population density (>400 people per km^2^), relatively homogenous settlement patterns which have evolved over many centuries, and the historical network configuration which most efficiently meets this settlement landscape. Since the utility companies’ customer demand numbers at asset levels are not publicly published or made available, often citing security reasons, the Voronoi tessellation-based approach gives reasonable customer demand estimates. The railway customer numbers are based on a flow assignment model that uses available train timetable data to map routes for railway journeys and station usage statistics to infer the number of people travelling along the routes. The third column of Table S-I describes the customer demand allocation models and data assembled for this study.

Table S-I: Description of the network data used in the study

| **Sector** | **Spatial and Topological Attributes** | **Customer demand allocation** |
| --- | --- | --- |
| Electricity Distribution   - Spatial coverage: South-East England - Voltage: 132 kV - Nodes: 253 | Nodes derived from Ordnance Survey (2015) and UK Power Networks (2014) | Estimated using Voronoi decomposition – detailed in papers (Pant, Thacker, Hall, Alderson, & Barr, 2017; Thacker, Barr, Pant, Hall, & Alderson, 2017) |
| Fresh-water towers   - Spatial coverage: South-East England - Nodes: 653 | Obtained from Ordnance Survey (2015) | Estimated using Voronoi decomposition – detailed in papers (Pant et al., 2017; Thacker et al., 2017) |
| Waste-water treatment   - Spatial coverage: South-East England - Nodes: 370 | Obtained from Ordnance Survey (2015) | Estimated using Voronoi decomposition – detailed in papers (Pant et al., 2017; Thacker et al., 2017) |
| Macrocellular basestations   - Spatial coverage: South-East England - Nodes: 1043 | Obtained from Ordnance Survey (2015) | Estimated using Voronoi decomposition – detailed in papers (Pant et al., 2017; Thacker et al., 2017) |
| Railways   - Spatial coverage: Great Britain - Nodes: 4492 stations and junctions - Edges: 4999-line route elements | Obtained from Ordnance Survey (2015) | Railway passenger trip allocations based on model described in (Pant, Hall, & Blainey, 2016), which uses train timetable data (Association of Train Operating Companies, 2015) and station usage statistics (Office of Rail and Road, 2015). |

Figure S-1 shows the GIS map representation of all the node assets (excluding railways) in the London, South East and East of England region of this study.

Figure S-1: GIS representation of the point assets and the count of the number of different infrastructure asset types


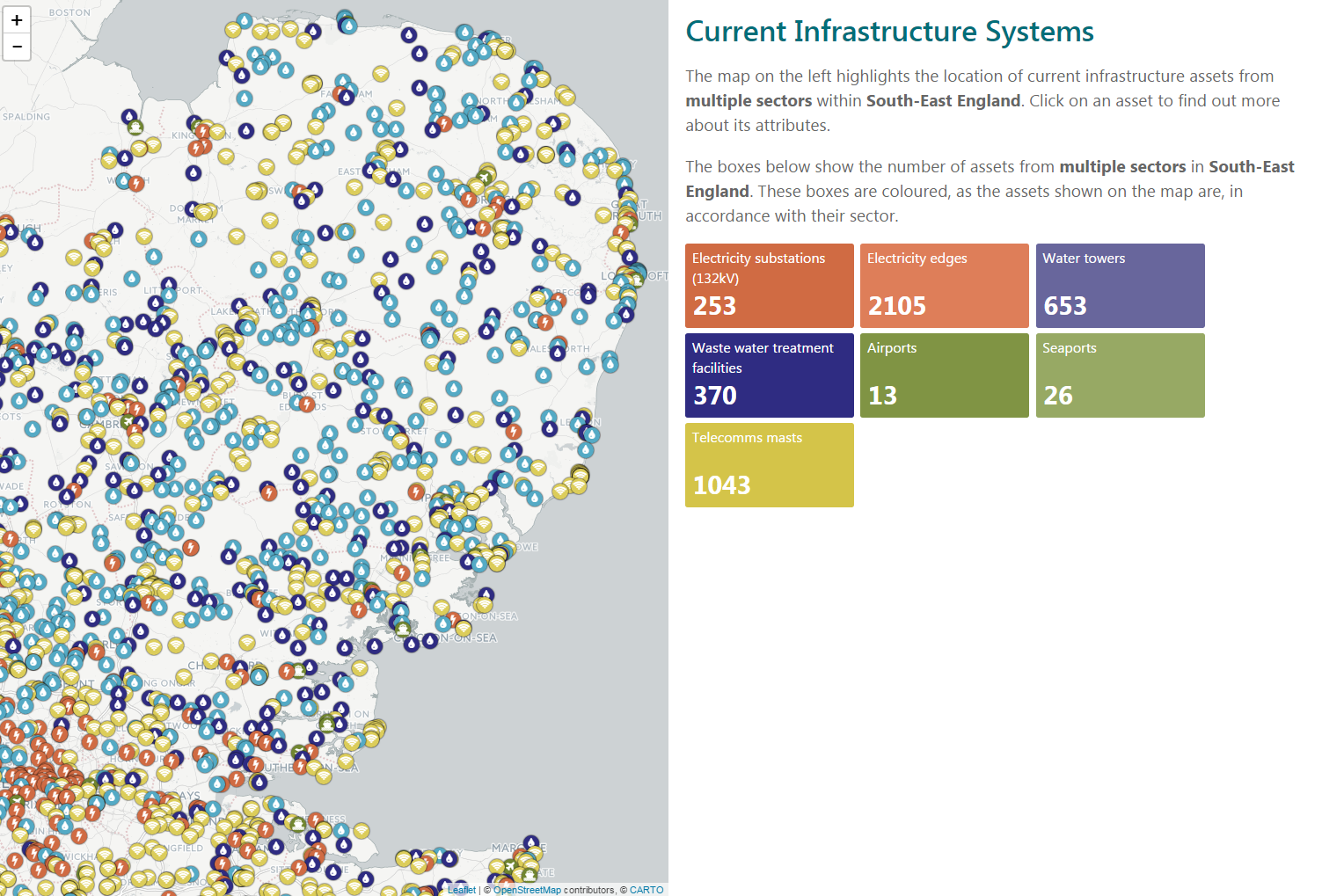

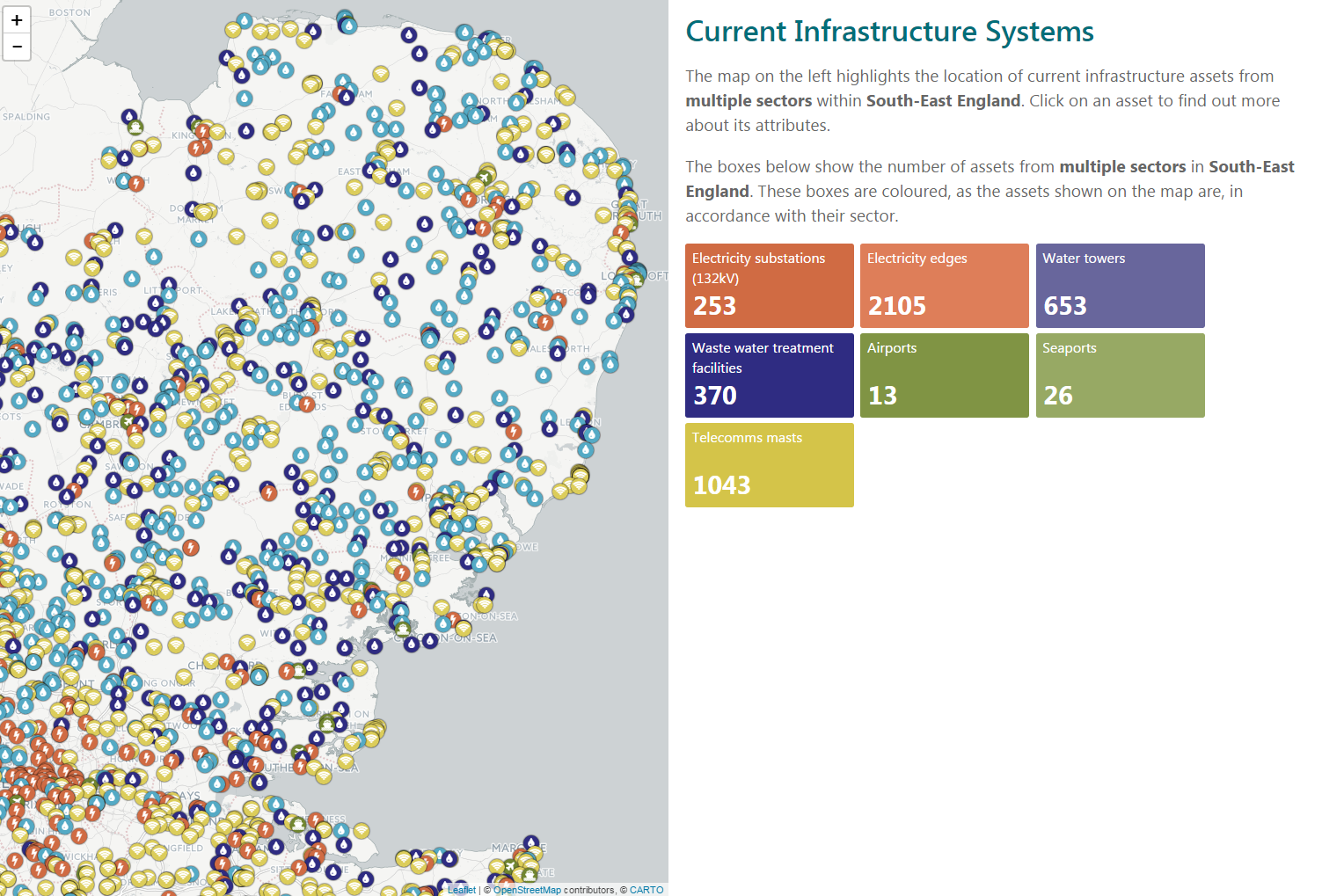


The dependency relationship between electricity and other infrastructures is inferred by either: (1) assembling information through the Ordnance Survey data, of some instances where electricity cables and overhead lines are shown to be connecting electricity and other assets; or (2) identifying the nearest substation to an infrastructure asset and connecting them via a ‘straight line edge’ to infer dependent relationship. Such radial connectivity of the 132 kV electricity substations to nearest assets is also reflective of the real-world connectivity between such systems in the UK. In the evolution of the electricity networks in the UK, 132kV substations represent grid-supply points that connect the 400 kV or 275 kV electricity transmission network to the lower level 33kV, 11 kV or 400/230 volts electricity distribution networks and other infrastructures (Parliamentary Office for Science and Technology, 2001). Generally, these grid-supply point serve a unique area and collection of assets, where they are the only point of connection between the transmission and lower level distribution networks. Figure S-2 shows an example of the connectivity between selected electricity substations and other infrastructure assets at the local level for Cambridge, UK.

As shown in Figure S-2, 2105 electricity dependency network edges are created in the analysis.

Figure S-2: Visual representation of example dependencies with respect to electricity for other infrastructures
